# Feasibility of training community health workers to conduct periodontal examinations: a validation study in rural Nepal

**DOI:** 10.1186/s12913-020-05276-5

**Published:** 2020-05-11

**Authors:** Daniel J. Erchick, Nitin K. Agrawal, Subarna K. Khatry, Joanne Katz, Steven C. LeClerq, Bhola Rai, Mark A. Reynolds, Luke C. Mullany

**Affiliations:** 1 Department of International Health, Johns Hopkins Bloomberg School of Public Health, 615 N. Wolfe St, Baltimore, MD 21205 USA; 2 Department of Dentistry, Institute of Medicine, Tribhuhvan University, Maharajgunj, P.O. Box 152, Kathmandu, Nepal; 3 Nepal Nutrition Intervention Project—Sarlahi (NNIPS), Kathmandu, Nepal; 4 Department of Advanced Oral Sciences and Therapeutics, University of Maryland School of Dentistry, 650 W. Baltimore Street, 4th floor, Suite 4222, Baltimore, MD 21201 USA

**Keywords:** Nepal, Oral health, Validity, Validation, Community-based

## Abstract

**Background:**

In many low- and middle-income countries, insufficient human resources limit access to oral health services. Shifting clinical tasks to less specialized health professionals, such as community health workers, has been used as a strategy to expand the health workforce, especially in remote or underserved locations. The objective of this study was to evaluate the validity of periodontal examinations conducted by auxiliary nurse midwives in a rural home setting in Nepal.

**Methods:**

Twenty-one pregnant women < 26 weeks gestation from Sarlahi District, Nepal, underwent full mouth periodontal examinations measuring probing depth (PD) and bleeding on probing (BOP) on 6 sites per tooth by one of five auxiliary nurse midwives, who were trained for this study but had no previous training in dentistry. After a 15-min break, each participant was examined again by an experienced dentist. Measures of validity for PD and BOP were calculated comparing the pooled and individual auxiliary nurse midwives to the dentist. A multivariable GEE model estimated the effect of periodontal characteristics on agreement between the auxiliary nurse midwives and the dentist.

**Results:**

Participant mean age was 22 years (SD: ±3 years), mean PD was 1.4 mm (SD: 03 mm), and 86% of women had BOP (according to the dentist). Percent agreement, weighted kappa scores, and intraclass correlation coefficients for PD, with an allowance of ±1 mm, exceeded 99%, 0.7, and 0.9, respectively, indicating an acceptable level of agreement. Auxiliary nurse midwives tended to report higher PD scores relative to the dentist, although this over-estimation was small and unlikely to impact population-based estimates of important indicators of oral health status. GEE regression modeling indicated similar agreement for mandible vs. maxilla, left vs. right side, and PD (≤2 mm, > 2 mm), and lower agreement for posterior teeth and lingual and proximal sites.

**Conclusion:**

Auxiliary nurse midwives were able to accurately conduct periodontal examinations in a rural home setting, suggesting the potential to shift tasks away from highly trained dentists and periodontal examiners in low-resource communities.

**Trial registration:**

ClinicalTrials.gov Identifier: NCT01177111 (Nepal Oil Massage Study); registered on August 6th, 2010.

## Background

Oral diseases constitute a major burden of chronic disease, collectively impacting half of the global population [1]. Leading causes of oral disease include dental caries, which affect a third of the population; gingivitis, a highly prevalent condition in children and adults; and severe periodontitis, a major cause of tooth loss, found in 10 to 15% of adults [2]. Disadvantaged communities, in both high- and low-income countries, have higher rates of oral disease and more limited access to oral health care, especially preventative services [3]. Critical knowledge gaps about the inequities in oral health care exist, including those related to the implementation of effective community-based intervention programs for oral diseases [4, 5].

In Nepal, like many low-income countries, oral health facilities, equipment, and qualified personnel, are in short supply [6]. Nepal has only two dentists per 100,000 people, one of the lowest ratios among South Asian countries [7]. In such contexts, shifting clinical tasks to less specialized health care workers may help alleviate the human resource demand for research or programmatic purposes [8]. Community health workers (CHWs) have demonstrated the ability to safely and effectively conduct clinical diagnostic tasks, and deliver essential care, for a variety of diseases in low resource settings [9–11]. Yet fewer studies have evaluated the ability of CHWs to adopt oral health services, with most previous investigations focused on oral health promotion; diagnostic screening, typically for childhood caries; or, in limited cases, providing simple preventative services. Shifting other aspects of preventative or therapeutic clinical oral health care to CHWs, such as screening for periodontal diseases, have not been evaluated [12].

Periodontal assessment is an important component of routine oral health care for diagnosis and management of common conditions in adults like gingivitis and periodontitis. Periodontal disease has also been implicated as a risk factor for chronic diseases, including adverse pregnancy outcomes and cardiovascular disease [13–16]. Shifting responsibility for periodontal assessment to CHWs would require assessment of the validity of procedures prior to wider implementation, which is especially important considering the precise techniques involved in various clinical periodontal measurements.

Given the potential benefit of extending access to periodontal examination in community settings in low-resource areas, we estimated the validity of periodontal measurements collected by auxiliary nurse midwives relative to an experienced dentist in rural Sarlahi, Nepal.

## Methods

We recruited twenty-one pregnant women < 26 weeks gestation in a sub-area of Sarlahi District, Nepal, between January and November 2016. These women were enrolled in a community-based, prospective cohort study of maternal gingival inflammation and adverse pregnancy outcomes. Participants in this study were identified and determined eligible using the infrastructure of a large community-based randomized trial, the Nepal Oil Massage Study (NOMS) (NCT01177111), which was actively enrolling a population-based sample of pregnant women in Sarlahi District.

Five female auxiliary nurse midwives, all of whom had completed an 18-month government certified midwifery program and resided in the study area, were selected as data collectors for the study. The auxiliary nurse midwives had no previous training or professional experience in dentistry or clinical research. A training course for the auxiliary nurse midwives was designed and conducted by an experienced dentist (NA) from the Department of Dentistry, Institute of Medicine, Tribhuvan University, Kathmandu, Nepal. The training course covered basic dental anatomy, oral pathology, and the procedures for periodontal examination. Practical training included identification of plaque and calculus, signs of gingivitis, and measurement of probing depth (PD), bleeding on probing (BOP), and distance from the cemento-enamel junction to the free gingival margin (CEJ-GM). Auxiliary nurse midwives were also trained in clinical research methods and ethics for human subjects research. Training lasted 3–4 weeks and covered both classroom instruction and practice of periodontal techniques under the guidance of the dentist.

**Fig. 1:**
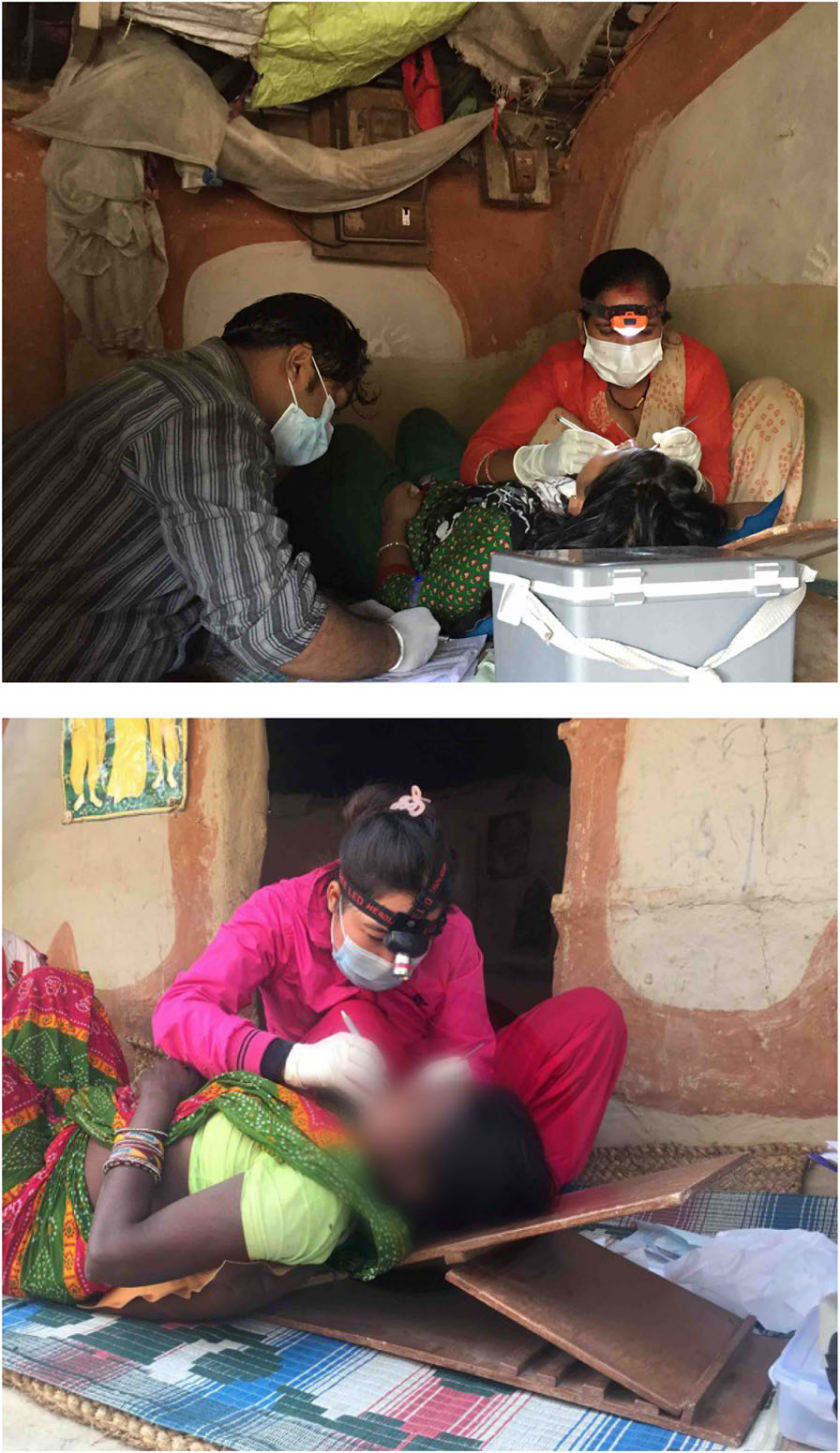
Auxiliary nurse midwives conduct periodontal examinations on study participants at their homes in a rural area of Sarlahi District in the Terai region of Nepal

All study visits were conducted at participant’s homes (Fig. 1) because of the wide dispersion of households and villages across this rural community and the impracticality of bringing participants to a central location. Auxiliary nurse midwives and an assistant traveled to participants’ homes by motorbike, carrying with them all of the equipment required to conduct the exam. After the consent process, auxiliary nurse midwives asked the participant where in the home they felt most comfortable having the examination. A location was selected by the auxiliary nurse midwives to conduct the examination that maximized the available natural light and participant privacy. Often examinations were conducted inside the house on a bed or the floor in dim lighting. Ideal conditions were found in households with an enclosed courtyard, allowing for the exam to be conducted outdoors on the porch or ground, providing the most natural light. Electric lighting was present in very few households, and whether examinations were conducted inside or outside, auxiliary nurse midwives relied on battery-powered headlamps to illuminate the mouth.

Auxiliary nurse midwives conducted a full mouth examination on each of the participants in the household. After the examination, participants rinsed their mouth with water and rested for fifteen minutes. Finally, the dentist, who was masked to the results of the auxiliary nurse midwives, conducted a second examination in the household under the same conditions. Data were recorded on paper forms by a trained assistant and electronically entered by experienced data entry operators. Periodontal measurements were made using a color Williams probe (Hu-Friedy, Chicago, IL, USA). PD was measured on six sites per tooth (disto-, mid-, and mesial- aspects of buccal and lingual surfaces) and the CEJ-GM distance on two sites per tooth (mid- buccal and lingual aspects), excluding third molars. After probing each quadrant, the presence or absence of BOP was recorded for buccal and lingual surfaces of each tooth. PD values were recorded in millimeters from 1 to 10, rounded to the next higher whole number. CEJ-GM distances were recorded similarly, with values of 0 to 10 mm. If the free gingiva was coronal to the CEJ, the CEJ-GM measurement was recorded as 0. Clinical attachment loss (CAL) was calculated by summing PD and the CEJ-GM distance; the CEJ-GM distance was assigned a value of 0 for distal and mesial sites, where this measure was not collected.

After the examination, auxiliary nurse midwives had a conversation with participants to provide basic information on oral health, covering messages about oral self-care, cessation from smoking, chewing tobacco, and use of betel nut, and care-seeking for preventative and therapeutic services. Messages on care seeking included sharing a list of the nearest government-run dental health care facilities. Participants with signs of periodontal disease, or other potential conditions identified by the auxiliary nurse midwives during the examination, were encouraged to improve their oral hygiene and seek care from one of these facilities.

Basic participant characteristics were reported as percentages. Differences between periodontal characteristics, as measured by the auxiliary nurse midwives and the dentist, were evaluated using paired t-tests or McNemar’s chi-squared tests. Percent agreement was calculated between the pooled auxiliary nurse midwives and each individual worker compared to the dentist for perfect agreement and considering values of PD ±1 mm as agreement. Confidence intervals (95%) for percent agreement were adjusted for clustering associated with the measurement of multiple teeth per participant using a generalized estimating equation (GEE) model. Kappa and weighted kappa (considering PD ±1 mm as agreement) statistics for PD were calculated, using tooth site as the unit of analysis, for pooled and individual auxiliary nurse midwives relative to the dentist. Confidence intervals (95%) for weighted and unweighted kappa statistics were also adjusted for clustering by participant using a bootstrap approach (1000 replications). Similarly, intraclass correlation coefficients (ICC) and associated cluster-adjusted, bootstrapped 95% confidence intervals were calculated for PD, using absolute agreement and tooth site as the unit of analysis, comparing the auxiliary nurse midwives to the dentist for perfect agreement and PD ±1 mm agreement. Sensitivity and specificity for the classification of tooth sites as PD > 2 mm were calculated for pooled and individual auxiliary nurse midwives vs. the dentist. A GEE model was used to estimate the effect of periodontal characteristics on agreement between the pooled auxiliary nurse midwives and dentist.

This study received ethical approval from the Institutional Review Board at Johns Hopkins Bloomberg School of Public Health (Baltimore, USA) and the Ethical Review Board of the Nepal Health Research Council (Kathmandu, Nepal). Our study adheres to the Standards for Reporting Diagnostic accuracy studies (STARD) 2015 guidelines.

## Results

Twenty-one pregnant women < 26 weeks gestation were enrolled in this study. The mean age of participants was 22 (SD: ±3) years (Table 1). A majority of women (62%) were literate, 38% had no education, 38% 1–9 years of schooling, and 24% 10 or more years. Nearly three-quarters (71%) of participants lived in a house constructed from thatch, sticks, or bamboo, with the other 29% in a house of wood, brick, or stone, and half (48%) had no access to a latrine.

**Table 1:** Demographic characteristics

Characteristic	Frequency	Percent
**Age (years)**		
< 20	7	33.3
20–24	11	52.4
25–29	3	14.3
**Literacy**		
No	8	38.1
Yes	13	61.9
**Education (years)**		
0	8	38.1
1–9	8	38.1
≥ 10	5	23.8
**House construction material**		
None, thatch, sticks, or bamboo	15	71.4
Wood planks, brick, or stones with mortar	6	28.6
**Latrine**		
No latrine	10	47.6
Brick, concrete, or pit latrine	11	52.4

Demographic characteristics of study participants

**Fig. 2:**
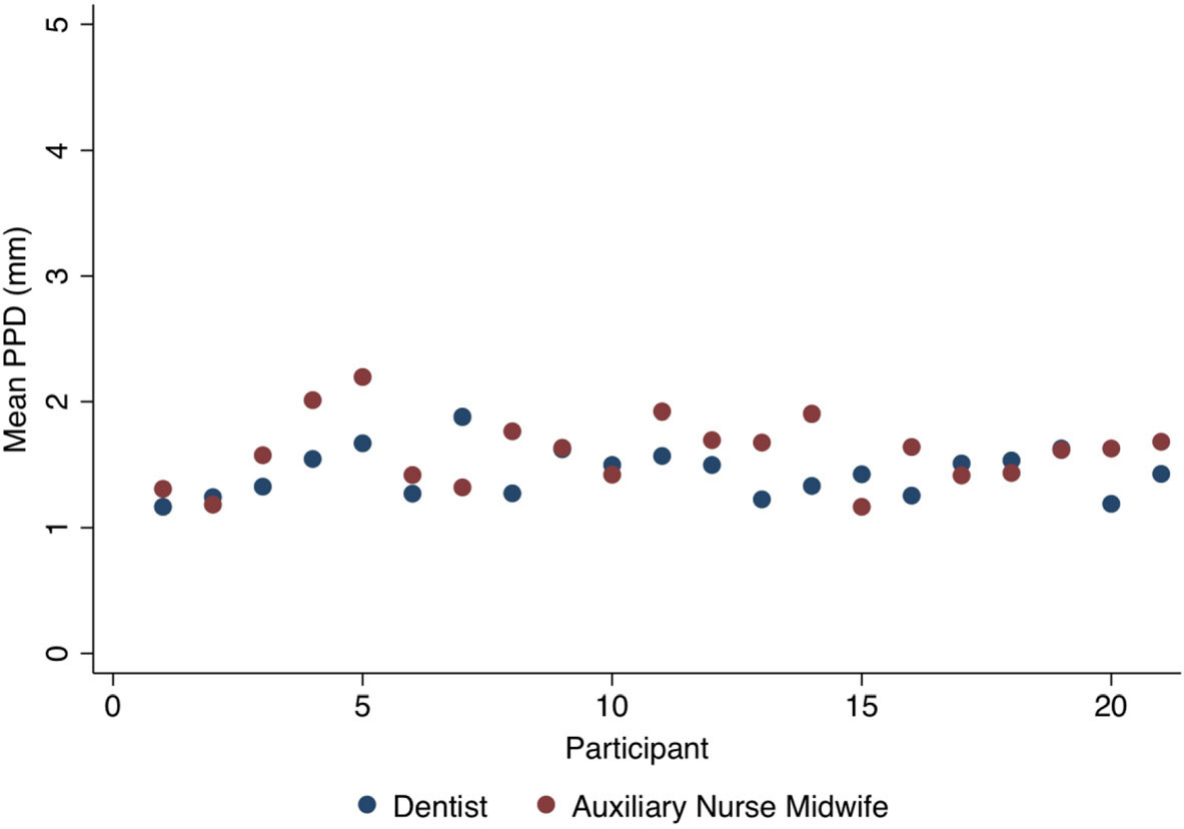
Mean probing depth (PD) measured by individual auxiliary nurse midwives and dentist for each participant. Comparison of mean probing depth (PD) by participant as measured by individual auxiliary nurse midwives and dentist

All participants had 28 teeth (excluding third molars), except for three women who were missing a single tooth (Table 2). Mean probing depth (PD) was 1.6 mm (SD: 0.3) with a range of 1 to 4 mm as measured by the auxiliary nurse midwives, and 1.4 mm (SD: 0.2) ranging 1 to 3 mm according to the dentist, a mean difference of 0.2 mm (*p* = 0.02) (Fig. 2). When non-congruent, these absolute differences in PD were nearly universally 1 mm, and roughly equally distributed in either direction. Collectively, the auxiliary nurse midwives identified one woman from the total twenty-one participants with at least one site with PD ≥4 mm, although the dentist measured these sites as < 4 mm and identified no other women as having any sites with PD ≥4 mm.

**Table 2:** Periodontal characteristics

Characteristic	Auxiliary nurse midwives	Dentist	*P*-value
**Number of teeth**^a^			
Mean number of teeth	27.9 ± 0.36	27.9 ± 0.36	–
**Bleeding on probing (BOP)**			
Proportion of sites BOP	13.6 ± 11.3	11.4 ± 12.0	0.22
≥ 1 site BOP (No. (%))	18 (85.7)	17 (81.0)	0.56
≥ 1 site & < 10% of sites BOP (No (%))	5 (23.8)	8 (38.1)	–
≥ 10% & < 25% of sites BOP (No (%))	8 (38.1)	5 (23.8)	–
≥ 25% of sites BOP (No (%))	5 (23.8)	4 (19.1)	0.56
**Probing depth (PD)**			
Mean PD (mm)	1.6 ± 0.3	1.4 ± 0.2	0.02
Proportion of sites with PD ≥3 mm	9.3 ± 11.5	4.2 ± 6.0	0.05
≥ 1 site PD ≥4 mm (No (%))	1 (4.8)	0 (0.0)	–
**Clinical attachment loss (CAL)**^b^			
Mean CAL (mm)	1.6 ± 0.3	1.4 ± 0.2	0.02
≥ 1 site CAL ≥4 mm (No (%))	2 (9.5)	1 (4.8)	0.56

Data presented as mean ± SD unless otherwise noted

^a^ Excluding third molars

^b^ Direct site only

Periodontal characteristics of study participants as measured by the auxiliary nurse midwives and dentist

The mean number of sites with bleeding on probing (BOP) was 13.6 (SD: ±11.3) and 11.4 (SD: ±12.0) for the auxiliary nurse midwives and the dentist, respectively (*p* = 0.22). Most women, 86% according to the auxiliary nurse midwives, and 81% according to the dentist, had at least some bleeding (*p* = 0.56). The auxiliary nurse midwives identified two, and the dentist three, of 21 total participants as having ≥1 mm recession of the gingival margin from the CEJ. Therefore, mean clinical attachment loss (CAL) did not differ substantially from the measures of PD.

Overall percent agreement of PD for the auxiliary nurse midwives relative to the dentist was 63% (95% CI: 58, 69%) for perfect agreement and 99% (95% CI: 99, 100%), for ±1 mm agreement (Table 3). Percent agreement differed significantly (*p* < 0.001) when stratified by PD, ranging from 62% agreement for PD = 1 mm to 48% for PD = 3 mm. For the individual auxiliary nurse midwives vs. the dentist, percent agreement ranged from 58% (95% CI: 44, 72%) to 69% (95% CI: 54, 83%) (Additional File 1) and percent agreement ±1 mm ranged from 99% (95% CI: 97, 100%) to 100% (Table 4). We calculated a design effect of 13.2 for the perfect agreement among the pooled auxiliary nurse midwives, indicating a high level of variation in PD between subjects. By individual participant, percent agreement and agreement ±1 mm ranged from 40 to 90% and 96 to 100%, respectively. Design effects for perfect percent agreement among the individual auxiliary nurse midwives ranged from 3.7 to 25.9.

**Table 3:** Intraclass correlation coefficients and kappa statistics for pooled auxiliary nurse midwives vs. dentist

Measure	Point estimate	95% CI
Percent agreement	63.3	57.5, 69.1
Percent agreement ±1 mm	99.3	98.8, 99.8
ICC	0.43	0.35, 0.51
ICC ±1 mm	0.94	0.91, 0.98
Kappa	0.32	0.24, 0.41
Kappa ±1 mm	0.85	0.76, 0.93

Validity measures comparing pooled auxiliary nurse midwives to the dentist

**Table 4:** Measures of probing depth agreement and sensitivity and specificity of probing depth (PD) > 2 mm and bleeding on probing (BOP) classification for individual auxiliary nurse midwives vs. dentist

			PD agreement			PD > 2 mm^b^				BOP^b^	
ANM	N^a^	Tooth sites	Percent agreement ± 1 mm	ICC ± 1 mm	Kappa ± 1 mm	No. sites	Sensitivity	Specificity	No. sites	Sensitivity	Specificity
1	4	669	100.0 (99.5, 100.0)	1	1	9	55.60%	95.30%	126	71.40%	89.00%
2	4	666	98.8 (97.7, 99.9)	0.92 (0.83, 1.00)	0.85 (0.65, 1.00)	24	83.30%	91.50%	78	30.80%	95.90%
3	4	666	98.8 (97.4, 100.0)	0.90 (0.80, 1.00)	0.74 (0.67, 0.78)	27	38.10%	94.70%	54	66.70%	87.80%
4	5	834	99.3 (98.3, 100.0)	0.93 (0.85, 1.00)	0.79 (0.69, 0.95)	33	18.50%	98.60%	108	41.70%	91.30%
5	4	672	99.7 (99.4, 100.0)	0.97 (0.95, 1.00)	0.92 (0.79, 1.00)	13	84.60%	97.00%	30	60.00%	93.50%

Data presented as point estimate (95% CI)

^a^ Number of participants assessed by both the auxiliary nurse midwives and dentist

^b^ Number of sites with PD > 2 mm and BOP according to dentist’s measurement

Validity measures comparing individual auxiliary nurse midwives to the dentist for probing depth ± 1 mm agreement and sensitivity and specificity of probing depth (PD) > 2 mm and bleeding on probing (BOP) classification

The kappa score for PD agreement between the pooled auxiliary nurse midwives and the dentist was 0.32 (95% CI: 0.24, 0.41) for perfect agreement, and 0.85 (95% CI: 0.76, 0.93) for agreement within ±1 mm. Kappa scores for the five individual auxiliary nurse midwives compared to the dentist ranged from 0.24 (95% CI: 0.06, 0.42) to 0.40 (0.22, 0.59) for perfect agreement, and 0.74 (95% CI: 0.67, 0.78) to 1.0 for agreement within ±1 mm. The intraclass correlation coefficient (ICC) between the pooled auxiliary nurse midwives and the dentist was 0.43 (95% CI: 0.35, 0.51) for perfect agreement, and 0.94 (95% CI: 0.91, 0.98) for agreement within ±1 mm. ICC values for the five individual auxiliary nurse midwives compared to the dentist ranged from 0.34 (95% CI: 0.16, 0.55) to 0.49 (95% CI: 0.31, 0.66) for perfect agreement, and 0.90 (95% CI: 0.80, 1.00) to 1.0 for agreement within ±1 mm.

Relative to the dentist, the pooled auxiliary nurse midwives classified individual tooth sites as PD ≤2 mm or PD > 2 mm with 50% sensitivity and 96% specificity. For the five individual auxiliary nurse midwives, each relative to the dentist, sensitivity ranged from 19 to 85%, and specificity from 92 to 99%. However, auxiliary nurse midwives classified more sites as PD > 2 mm than the dentist, an average of 5.6% sites per participant vs. 2.5%. Sensitivity and specificity of BOP were 53.8 and 91.5%, respectively, for the pooled auxiliary nurse midwives relative to the dentist, with sensitivity ranging from 31 to 71%, and specificity from 88 to 96%, for individual auxiliary nurse midwives.

We modeled the relative risk (RR) of percent agreement for PD of the auxiliary nurse midwives relative to the dentist using a generalized estimating equation (GEE) regression model, including covariates for periodontal characteristics (Table 5). Covariates for jaw (maxilla, mandible), side (left, right), and probing depth (≤2 mm, > 2 mm) were not significantly related to percent agreement. PD measurements on posterior teeth and lingual surfaces were associated with an average reduction in percent agreement of 15% (RR 0.85, 95% CI: 0.81, 0.90) and 10% (RR 0.90, 95% CI: 0.86, 0.95), respectively. Measurements on direct tooth site, relative to the proximal site, were associated with a 21% increase in agreement.

**Table 5:** Relationships between probing depth (PD) agreement and periodontal characteristics using a multivariable GEE model

Variables	RR	95% CI
Jaw		
Mandible	0.96	0.92, 1.00
Maxilla	Ref	–
Side		
Right	1.01	0.97, 1.06
Left	Ref	–
Position		
Posterior	0.85	0.81, 0.90
Anterior	Ref	–
Surface		
Lingual	0.90	0.86, 0.95
Facial	Ref	–
Site		
Direct	1.21	1.15, 1.28
Proximal	Ref	–
Probing depth		
> 2 mm	0.85	0.70, 1.05
≤ 2 mm	Ref	–

Multivariable GEE model reporting relative risk (RR) of PD agreement between auxiliary nurse midwives relative to the dentist by periodontal characteristics

## Discussion

Our results demonstrate that auxiliary nurse midwives with minimal training can satisfactorily conduct periodontal examinations at patient homes in rural Nepal. Percent agreement, weighted kappa scores, and intraclass correlation coefficients, with an allowance of ±1 mm, exceeded 99%, 0.7, and 0.9, respectively, indicating an acceptable level of agreement. While the auxiliary nurse midwives tended to overestimate probing depth (PD) scores relative to the dentist, the magnitude of over-estimation was small (0.2 mm) and unlikely to impact population-based estimates of critical indicators. Relative to the dentist, the capacity of the auxiliary nurse midwives to distinguish sites with PD > 2 mm was less than ideal (sensitivity of 50%); however, auxiliary nurse midwives demonstrated an excellent ability to discern sites with PD ≤2 mm (specificity 96%). Sensitivity and specificity for bleeding on probing (BOP) data exhibited a similar pattern to PD, but should be interpreted with caution, due to the possible influence of the first examination on the subsequent one, given the short recovery interval (15 min) for each participant.

Globally, studies have shown that CHWs can safely and effectively deliver a range of health promotion services, diagnostic screenings, and therapeutic interventions in community-based, low-resource settings. These include, for example, ultrasound examination for diagnosis of obstetric risk factors, sign-based algorithms for assessment of omphalitis, and pneumonia case management [9, 10, 17]. However, the literature on training of CHWs to provide oral health care services is less well-developed relative to other areas, and is primarily limited to health promotion and use of simple diagnostic screening tools [12].

Available evidence from LMICs supports the ability of CHWs to conduct oral health promotion activities to teach proper oral hygiene behaviors, commonly brushing and flossing, and encourage care seeking. In the city of Rio Grande da Serra in southeastern Brazil, a pre-post intervention study showed that a training program and regular supervision for CHWs from a family health program led to improved oral health knowledge, oral hygiene behaviors, and care seeking and utilization of oral health services of women in the community [18]. Another pre-post intervention study, conducted in north Indian city of Chandigarh, reported that an oral hygiene education package delivered by local community-based Anganwadi workers, who are responsible for maternal and child health education, led to a decrease in plaque and caries activity among children and an increase in self-reported oral hygiene behaviors [19].

A few studies have shown that CHWs in LMICs can utilize oral health diagnostic screening tools in community-based settings, including to identify childhood caries or oral disease in the elderly. A study in Brazil found use of the Revised Oral Assessment Guide by community health workers, relative to a dentist, to have acceptable validity and reliability for the identification of oral health problems in elderly individuals in a clinic setting [20, 21]. In Thailand, CHWs, who had a secondary school level education, were successfully trained to provide oral health education, conduct root scaling and planing, sterilize instruments, control infection, and make referrals [22]. In Australia, several studies evaluated efforts to train Aboriginal Health Workers to expand oral health promotion, diagnostic screening, and delivery of basic interventions to remote and rural areas of the country [23]. A cluster randomized trial in Australia’s Northern Territory reported that health promotion and application of fluoride varnish by Aboriginal Health Workers reduced dental caries in young children [24].

Findings from this study suggest that auxiliary nurse midwives, who far outnumber dentists and other oral health workers in Nepal, have the potential to expand access to oral health services in the community. Auxiliary nurse midwives and other similar CHWs live nearby and work in health facilities at all levels of government across Nepal, including in rural areas where access to oral health care is generally lower. In 2018, there were 31,764 auxiliary nurse midwives registered with the Nepal Nursing Council, although staffing shortages for auxiliary nurse midwives exist across the health system [25]. For example, in 2015, the percent of sanctioned health posts for auxiliary nurse midwives that were filled ranged between 71 to 79% for different types of facilities from the zonal to the health post levels, with the exception of urban health centers, which were staffed at 36% capacity [26].

Beyond availability of auxiliary nurse midwives and similar CHWs, other issues would need to be considered by any effort to shift responsibility for delivery of oral health services to this cadre of CHWs. A few such questions include how effective oral health information provided by CHWs is at changing oral hygiene behaviors and reducing oral disease in this community; whether CHWs can safely and effectively provide basic oral health treatments in a community setting; how CHWs would refer patients requiring more skilled care to nearby dental health facilities; and whether delivery of oral health services by CHWs in rural communities provides cost savings to the health system.

Periodontal examination in a community-setting presents various challenges not found in a typical clinical setting, including difficult field conditions, such as the absence of a high-quality light source [27]. Some variability observed in the measures of validity in this study may be attributable to the field conditions. We identified lower agreement on posterior teeth, lingual surfaces, and proximal sites, areas that may be more difficult to measure accurately in low light. Alternatively, this variability could have resulted from the limited training of the auxiliary nurse midwives, or even normal inter-rater variability, as lower agreement for posterior, lingual, and proximal sites has also been seen in reliability studies utilizing highly trained periodontal examiners [28, 29].

A majority of women in our study had signs of gingival inflammation, but few had significant probing depths or attachment loss. This could be a result of the young age of the study population, which, at a mean of 22, is low even for studies of the periodontal disease and adverse pregnancy outcome association [30]. Alternatively, the small sample size of this study may have played a role; data from the parent cohort study indicate a prevalence of ≥1 site with PD ≥4 mm of over 8% [31]. As a result of the absence of moderate and severe disease, the bulk of our analyses focused on discriminating between low probing depth scores (i.e., 1–2 mm), which are indicative of periodontal health. This limited our ability to fully explore the capacity of the auxiliary nurse midwives to accurately measure the full range of clinical periodontal parameters and distinguish between states of disease from health. Our assessment of the amount of measurement error associated with the study’s auxiliary nurse midwives may be underestimated if higher probing depths are measured with lower reliability in this setting as has been documented elsewhere [27, 32].

In recent years, periodontal researchers have sought to improve data quality by designing a standardized process to assess periodontal examiners [33–36]. Validity and reliability studies have been used to determine if periodontal measurements conducted by periodontal examiners are consistent with a gold standard or within and between examiners [37, 38]. Yet a review of the periodontal literature, conducted by Hefti and Preshaw (2012), showed that only 30% of publications using the Gingival Index or Modified Gingival Index reported on examiner assessment, and almost none discussed the possible consequences of examiner validity or reliability on the outcome of the study [37]. Studies of the periodontal disease and adverse pregnancy outcomes association often do not report on examiner assessment, and those that do typically include a limited mention in methods section without estimates of uncertainty or information on study design used to collect these data. Our estimates of validity were generally comparable to those documented by similar studies of the periodontal disease and adverse pregnancy outcome association, although some achieved both weighted kappas and ICCs of over 0.9 [39].

A limitation of this study was its small sample size, which was restricted for logistical reasons. With additional participants we could have estimated reliability measures of intra-rater agreement for the repeated measurements of each auxiliary nurse midwife and inter-rater agreement between the study’s five auxiliary nurse midwives. We prioritized validity over reliability because the auxiliary nurse midwives in this study had no previous experience in dentistry or clinical research. We could have also measured agreement using the subject as the unit of analysis, for example by classifying participants has having gingivitis or periodontitis, an approach that might have yielded more practical information for our parent study, which will take the subject as the primary unit of analysis in examining the relationship between periodontal disease and adverse pregnancy outcomes.

## Conclusion

Our results suggest the potential to shift delivery of certain basic oral health services from dentists and other highly trained professionals to auxiliary nurse midwives or similar CHWs. Policy and programmatic decisions to task CHWs with provision of oral health care should be taken in the context of the multiple factors that affect the success of community health worker programs, including health systems factors (e.g., human resource structures, management and supervision policies), community factors (e.g., health beliefs, community mobilization), and national socioeconomic and political factors (e.g., political will, poverty) [11]. Increased efforts are needed to consider how CHWs could expand access to essential oral health knowledge and services in low-resource settings.

## Supplementary information

**Additional file 1.** Intraclass correlation coefficients and kappa statistics for individual auxiliary nurse midwives vs. dentist with perfect agreement. Validity measures comparing individual auxiliary nurse midwives to the dentist for perfect probing depth agreement.

## Data Availability

All data files, codebooks, and related manuscripts are available from the JHU Data Archive (10.7281/T1/ZPGBJW).

## Abbreviations

BOP: Bleeding on probing
CEJ: Cemento-enamel junction
CAL: Clinical attachment loss
CHW: Community health worker
CI: Confidence intervals
GEE: Generalized estimating eq.
GM: Gingival margin
LMICs: Low- and middle-income countries
ICC: Intraclass correlation coefficient
NOMS: Nepal Oil Massage Study
PD: Probing depth
RR: Relative risk
SD: Standard deviation
