# Supplementary material for: Feasibility of training community health workers to conduct periodontal examinations: a validation study in rural Nepal
Source: BMC Health Serv Res. 2020 May 11;20:412. doi: 10.1186/s12913-020-05276-5 (PMC7212579; doi:10.1186/s12913-020-05276-5)
Supplement: Supplementary file 1 — Additional file 1. Intraclass correlation coefficients and kappa statistics for individual auxiliary nurse midwives vs. dentist with perfect agreement. Validity measures comparing individual auxiliary nurse midwives to the dentist for perfect probing depth agreement. [file 12913_2020_5276_MOESM1_ESM.docx]

**ADDITIONAL FILES**

**Additional File 1: Intraclass correlation coefficients and kappa statistics for individual auxiliary nurse midwives vs. dentist with perfect agreement**

| **Oral health worker** | **N^a^** | **Tooth sites** | **Percent agreement** | **ICC** | **Kappa** |
| --- | --- | --- | --- | --- | --- |
| 1 | 4 | 669 | 62.6 (52.7, 72.6) | 0.42 (0.32, 0.52) | 0.31 (0.17, 0.45) |
| 2 | 4 | 666 | 59.2 (53.1, 65.3) | 0.47 (0.32, 0.62) | 0.30 (0.20, 0.40) |
| 3 | 4 | 666 | 57.8 (44.1, 71.8) | 0.34 (0.16, 0.55) | 0.24 (0.06, 0.42) |
| 4 | 5 | 834 | 68.6 (54.0, 83.2) | 0.42 (0.29, 0.55) | 0.34 (0.15, 0.52) |
| 5 | 4 | 672 | 67.0 (55.5, 78.5) | 0.49 (0.31, 0.66) | 0.40 (0.22, 0.59) |
| Data presented as point estimate (95% CI) | | | | | |
| ^a^ Number of participants assessed by both the auxiliary nurse midwives and dentist | | | | | |
